# Innovative Approach to Pediatric Peutz–Jeghers Syndrome: Simultaneously Performing Laparoscopic Reduction of Polyp-Induced Intussusception and Polypectomy with Double-Balloon Endoscopy

**DOI:** 10.70352/scrj.cr.25-0194

**Published:** 2025-09-27

**Authors:** Naoko Mise, Yasushi Fuchimoto, Takahiro Shimizu, Nao Tanaka, Yoshitaka Shinno, Kentaro Yamashita

**Affiliations:** 1 Department of Pediatric Surgery, International University of Health and Welfare School of Medicine, Narita, Chiba, Japan; 2 Department of Gastroenterology, International University of Health and Welfare School of Medicine, Narita, Chiba, Japan

**Keywords:** Peutz–Jeghers syndrome, double-balloon enteroscopy, laparoscopy, intussusception, child, polypectomy

## Abstract

**INTRODUCTION:**

In patients with Peutz–Jeghers syndrome (PJS), repeated laparotomy and bowel resection can lead to short bowel syndrome and adhesions. Minimizing surgical invasiveness while effectively managing intussusception caused by polyps is therefore essential. We present a pediatric case of recurrent intussusception due to a jejunal polyp near the ligament of Treitz, successfully treated with a hybrid approach combining laparoscopic reduction and double-balloon enteroscopy (DBE)–assisted polypectomy.

**CASE PRESENTATION:**

A 9-year-old girl presented with small bowel intussusception and underwent laparoscopic-assisted resection of a 40-cm jejunal segment containing a 35-mm polyp and multiple adjacent polyps. Postoperative findings led to a diagnosis of PJS. Two weeks later, she developed recurrent intussusception. CT revealed a lead-point mass without signs of ischemia or strangulation. At reoperation, laparoscopic reduction was achieved except for residual intussusception near the ligament of Treitz. Under continuous laparoscopic observation, DBE identified a 40-mm polyp 30–40 cm distal to the pylorus. En bloc removal was not feasible; therefore, piecemeal resection was performed, resulting in complete release of the intussusception. Follow-up capsule endoscopy at 2 months confirmed no residual lesion.

**CONCLUSIONS:**

This case highlights the feasibility and safety of a hybrid laparoscopic–endoscopic approach for intussusception caused by proximal small bowel polyps in PJS, allowing bowel preservation and effective lesion removal. This strategy may serve as a valuable treatment option in selected cases.

## Abbreviations

DBE
: double-balloon enteroscopy
LADBE
: laparoscopically assisted DBE
PJS
: Peutz–Jeghers syndrome

## INTRODUCTION

PJS is an autosomal dominant disorder characterized by hamartomatous polyposis of the entire gastrointestinal tract except the esophagus, along with characteristic mucocutaneous pigmentation, mainly on the lips, mouth, and fingertips, caused by mutations in the LKB1/STK11 gene.^1)^ These polyps can cause anemia from chronic bleeding, impaired gastrointestinal transit, and intestinal intussusception; adhesions and medically induced short-bowel syndrome from multiple laparotomies are problematic.^2)^ Avoiding laparotomy and bowel resection in these patients is important for their management.

We reported here a case of recurrent intussusception in a 9-year-old girl with PJS. We discussed the efficacy of laparoscopic release of the intussusception and simultaneous polypectomy performed with DBE to avoid bowel resection. This manuscript was prepared following the CARE guidelines (https://www.care-statement.org).

## CASE PRESENTATION

A 9-year-old girl presented to our hospital with intermittent abdominal pain, vomiting, and bloody stools for the past 2 weeks. Upon arrival at our hospital, her abdomen was flat and soft, and the abdominal pain had disappeared. At the age of 4 years, she received a prescription for iron tablets from her family physician due to severe anemia. Her parents had no history of polyps or gastrointestinal cancer.

**Fig. 1:**
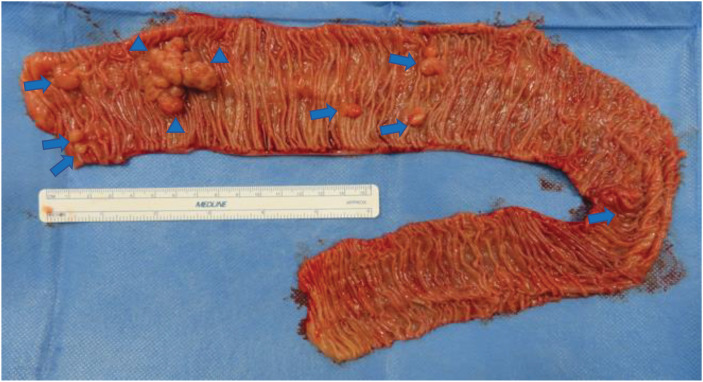
Resected specimen at the time of initial surgery. In the resected small bowel, a 35-mm sanguineous polyp and numerous small polyps are seen.

**Fig. 2:**
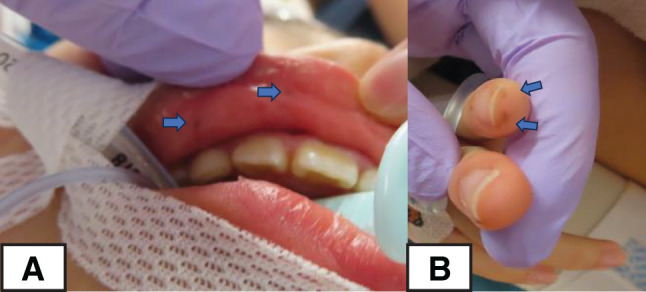
Skin findings at the time of initial surgery. (**A**, **B**) Small pigmented macules were seen on the lips, gingiva, and phalanges.

Ultrasonography and contrast-enhanced CT revealed a small bowel intussusception, and a laparoscopic-assisted small bowel resection was performed. A Lap Protector and EZ Access (Hakko, Nagano, Japan) were employed. A Lap Protector was placed through a small umbilical incision, and an EZ Access, equipped with three 5-mm trocars, was placed accordingly. The intussusception was released laparoscopically. Upon extracorporeal examination of the intussusceptum, several small tumors were palpated around the main tumor, which was located 290 cm from the ileocecal valve. Although most of the small intestine was palpated extracorporeally, no palpable tumors were found in the other areas. The 40 cm of the small intestine containing these palpable tumors was resected. The resected small intestine showed a 35-mm–sanguineous polyp and numerous small polyps (**Fig. 1**). Upon careful examination of the patient after surgery, small pigmented macules were seen on the lips, gingiva, and phalanges (**Fig. 2**). Together with the pathological findings, a diagnosis of PJS was made.

**Fig. 3:**
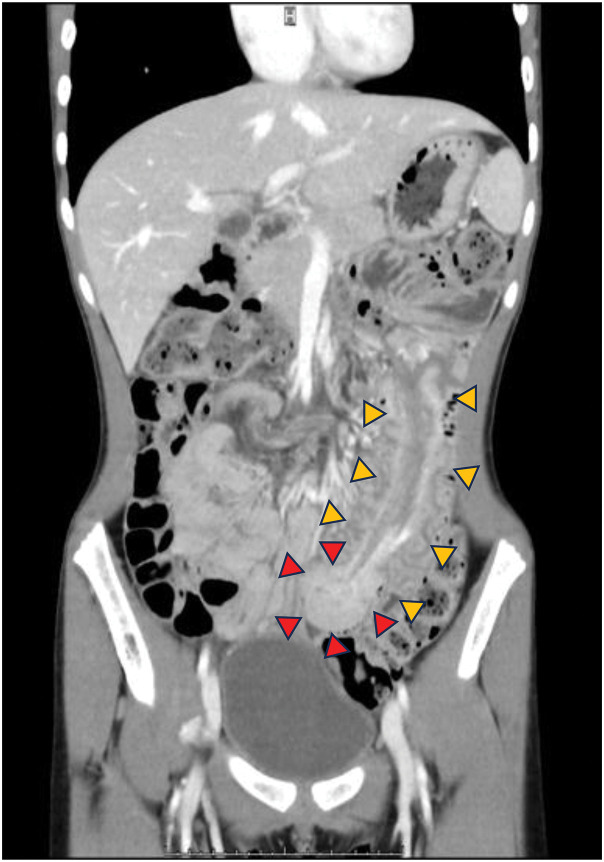
CT image of intestinal intussusception caused by a polyp. A CT scan showed an intussusception of the small bowel (yellow arrowheads) with a mass (red arrowheads) in the advanced region.

The endoscopy was scheduled for a later date, but 2 weeks after her discharge, she developed bilious vomiting that required emergency hospitalization. A contrast-enhanced CT scan revealed an intussusception of the small bowel from the left upper abdomen to the lower abdomen with a mass in the advanced region, but there were no findings consistent with intestinal ischemia or strangulated bowel obstruction (**Fig. 3**).

**Fig. 4:**
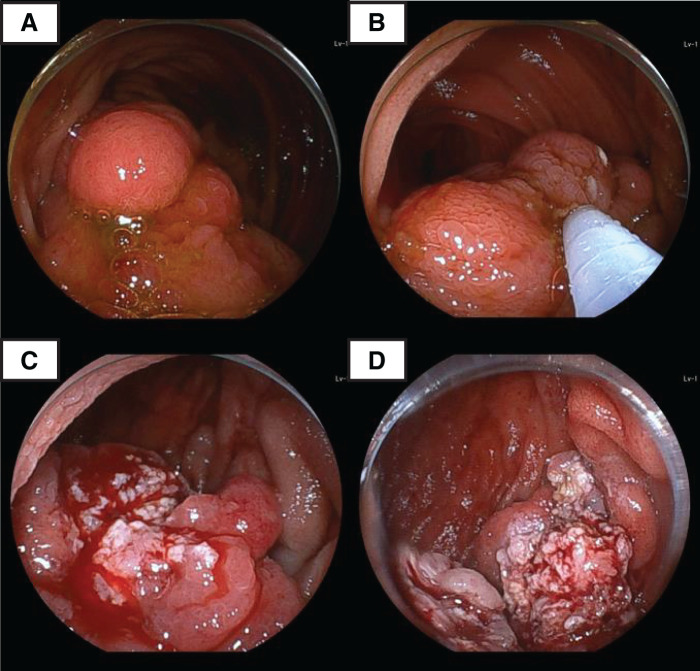
Endoscopic polypectomy. (**A**) A 40-mm subcordate polyp was seen in the jejunum, and its border with the small bowel mucosa was indistinct due to residual intussusception. (**B**–**D**) Piecemeal resection of the polyp was performed, and the intussusception was completely released.

The surgery was performed on the 2nd day of hospitalization. As in the previous surgery, the umbilicus was opened through an incision, and a Lap Protector and EZ Access with 2 ports were placed. A 5-mm port was also placed on the lower abdomen. Approximately 30 cm of intussusception was seen in the left lower abdomen and was released laparoscopically. In the left upper abdomen, adhesions were observed between the omentum and the mesentery and between the mesenteries, and the intestinal tract was erythematous and edematous. Due to chronic adhesions in the intussusceptum, most of the intussusception was released, though not completely. Under continuous laparoscopic observation, a double-balloon endoscope was inserted. There was a 40-mm subcordate polyp located in the ischemic 30–40 cm anally from the pylorus, and its border with the small bowel mucosa was indistinct due to residual intussusception. It was difficult to perform en bloc resection of the polyp, but piecemeal resection was performed, and the intussusception was released. Most of the polyp was eventually removed (**Fig. 4**). The resection site showed only oozing and did not require additional hemostatic treatment. The procedure was completed after laparoscopic confirmation of no perforation.

Upper and lower endoscopy and capsule endoscopy at a later date revealed only a few 2–5 mm polyps in the stomach, jejunum, ileum, and colon, but no polyps of any size that could have caused the intussusception.

## DISCUSSION

PJS is an autosomal dominant disorder, but approximately 50% of those who develop the disease have no family history.^3,4)^ In this case, there was no family history of PJS, and the characteristic pigmentation of PJS was small and not prominent, making PJS an unlikely diagnosis before the initial surgery. A laparoscopic reduction of the intussusception was successful, but a 40-cm bowel resection was necessary due to an intraoperative diagnosis of polyposis. Therefore, a more cautious strategy was needed to reduce surgical invasiveness and avoid bowel resection at the time of recurrence. We considered treatment with DBE alone, but due to the history of bowel resection and the possibility of unsuccessful release of intussusception with DBE, we decided to resect the polyp with DBE after laparoscopic release of intussusception.

In recent years, treating polyps endoscopically using balloon-assisted DBE has become possible, thus avoiding laparotomy. Ischemic treatment by implanting a clip or snare into the stalk of the polyp may have fewer side effects than polypectomy or endoscopic mucosal resection.^5)^ In cases of laparotomy or bowel resection, postoperative adhesions may reduce the insertability of the DBE, making endoscopic treatment difficult.

Stasinos et al. reported the laparoscopically assisted DBE (LADBE) technique using single incision laparoscopic surgery for patients with small bowel pathology who previously failed DBE.^6)^ LADBE in patients with PJS was also reported by Ross et al.^7)^ These reports describe the resection of polyps in patients with PJS that are difficult to reach or resect by endoscopy alone and do not mention cases of intestinal intussusception. There are a few reports of intussusception repaired by DBE.^8–10)^ Oguro et al. reported that endoscopic treatment was unsuccessful in only 1 of 25 cases. In this report, giant polyps required multiple ischemic treatments.^10)^

During the initial surgery, PJS was not suspected. However, multiple polyps were observed clustered around the lead point of the intussusception, prompting palpation of the entire small intestine to search for additional lesions. As a result, approximately 40 cm of small bowel, including the area of concern, was resected. Nevertheless, a more conservative resection limited to the base of the polyp, combined with postoperative removal of other lesions using DBE, might also have been an appropriate option. During the 2nd surgery, considering the patient’s age and the fixation of the bowel near the ligament of Treitz, we chose to avoid bowel resection, perform laparoscopic reduction of the intussusception as much as possible, and carry out piecemeal endoscopic resection of the polyp. While this approach avoided bowel resection, it raised concerns about whether complete removal had been definitively achieved. However, follow-up capsule endoscopy revealed no residual polyp at the treated site, supporting the effectiveness of this hybrid strategy. In future similar cases, more meticulous surgical planning will be required, considering both the extent of resection and the feasibility of an endoscopic approach.

The least invasive treatment is polypectomy after reduction of the intussusception by DBE alone. However, there are cases in which reduction of the intussusception by DBE is unsuccessful, and cases in which removal of the polyp is difficult even if the intussusception is reduced by DBE. It is unknown whether DBE can reach the lesion, especially in cases with a history of laparotomy or bowel resection, and if multiple DBEs are required to complete endoscopic polyp treatment, children have the disadvantage of having to undergo repeated general anesthesia.

If the DBE-first approach fails, especially in children with limited abdominal space, the difficulty in securing the visual field by endoscopic insufflation may make the transition to laparoscopic surgery difficult, necessitating open surgery. On the other hand, the use of laparoscopic observation in conjunction with the DBE procedure may provide a rapid response to problems such as perforation and bleeding associated with polypectomy. Our case suggests the utility of a hybrid technique combining DBE and laparoscopic surgery in such situations.

## CONCLUSIONS

The combination of laparoscopic intussusception release and endoscopic polypectomy may reduce surgical invasiveness and avoid bowel resection, which could be helpful for the treatment of intestinal polyposis in PJS.

## DECLARATIONS

### Availability of data and materials

The data that support the findings of this study are available from the corresponding author upon reasonable request.
